# Posterior-only surgical correction with heavy halo-femoral traction for the treatment of rigid congenital scoliosis associated with split cord malformation

**DOI:** 10.1186/s12891-020-3124-9

**Published:** 2020-02-13

**Authors:** Hong-Qi Zhang, Ang Deng, Ming-Xing Tang, Shao-Hua Liu, Yu-Xiang Wang, Qi-Le Gao

**Affiliations:** Department of Spine Surgery, Xiangya Hospital of Central South University, Xiangya Road 87, ChangSha, 410008 China

**Keywords:** Split cord malformation, Halo-femoral traction, Posterior-only, Rigid congenital scoliosis

## Abstract

**Background:**

Whether or not, prophylactic neurosurgical interventions of split cord malformation (SCM) before undertaking corrective surgery was the focus of debate. The present study was performed to evaluate the safety and efficacy of posterior-only surgical correction with heavy halo-femoral traction for the treatment of rigid congenital scoliosis (RCS) associated with SCM.

**Methods:**

From 2011 to 2017, 24 patients suffered from RCS associated with SCM underwent posterior-only surgical correction with heavy halo-femoral traction. The apex of the deformity was lumbar (*n* = 9), thoracic (*n* = 11), and thoracolumbar (*n* = 4). There were 13 cases of failure of segmentation; 4 cases of failure of formation and 7 cases of mixed defects. Based on SCM classification, there were 14 patients with SCM type 1 and 10 patients with SCM type 2. The Scoliosis Research Society (SRS)-22 and modified Japanese Orthopaedic Association (mJOA) scores were assessed preoperatively and at the final follow up.

**Results:**

The mean duration of surgery was 327.08 ± 43.99 min and the mean blood loss was 1303.33 ± 526.86 ml. The mean follow-up period was 20.75 ± 8.29 months. The preoperative mean coronal Cobb angle was 80.38° ± 13.55°; on the bending radiograph of the convex side, the mean Cobb angle was 68.91° ± 15.48°; the mean flexibility was 15.04% ± 7.11%. After heavy halo-femoral traction, the mean coronal Cobb angle was reduced to 56.89° ± 13.39°. After posterior-only surgical correction, postoperative mean coronal Cobb angle was further reduced to 32.54° ±11.33°. The postoperative mean correction rate was 60.51% ± 7.79%. At the final follow up, the corrective loss rate of Cobb angle was only 3.17%. The SRS-22 total score improved at the final follow-up evaluation compared with the preoperative SRS-22 total score. The spinal cord function was stable and there were no new neurological symptoms after correction. There were no significant differences between final follow-up and preoperative mJOA total scores.

**Conclusions:**

Without prophylactic neurosurgical intervention and spine-shortening osteotomy, posterior-only surgical correction with heavy halo-femoral traction could be safe and effective for the treatment of RCS associated with SCM.

## Background

Split cord malformation (SCM) is a rare type of congenital spinal cord abnormality however, it might be a common finding associated with congenital scoliosis (CS). Pang’s new nomenclature was established to avoid confusion between diplomyelia and diastematomyelia. In Type I SCM, the spinal cord is divided into two halves with two different dural sleeves by a fibrocartilaginous or bony spur. In Type II SCM, there is only a common intradural fibrous band and dural sac without bony spur [1, 2]. The treatment of CS associated with SCM is complex, and the spinal cord abnormality and spinal deformity must be taken into account. Prophylactic neurosurgical intervention of SCM before undertaking corrective surgery is the focus of debate in spine surgery and neurosurgery [3–5]. Conventional approach for management of CS associated with SCM was first to perform neurosurgical intervention for SCM and then to perform correction of scoliosis 3 to 6 months later. To date, there are no studies concerning the application of posterior-only surgical correction with heavy halo-femoral traction for the treatment of rigid congenital scoliosis (RCS) associated with SCM in the absence of prophylactic neurosurgical intervention and spine-shortening osteotomy. Therefore, the present study was performed to evaluate the safety and efficacy of posterior-only surgical correction with heavy halo-femoral traction for the treatment of RCS associated with SCM retrospectively.

## Methods

### General data

From 2011 to 2017, 24 patients (9 males and 15 females; age, 10–24 years; average age, 16.38 ± 4.56 years), suffered from RCS associated with SCM, were treated at our Department. In all patients X-ray, CT and MRI examinations revealed: the apex of the deformity was lumbar (*n* = 9), thoracic (*n* = 11), and thoracolumbar (*n* = 4); based on CS classification, all patients were divided into 3 types, including 13 cases of failure of segmentation, 4 cases of failure of formation, and 7 cases of mixed defects; based on SCM classification, all patients were divided into 2 types, including 14 cases of SCM type 1 and 10 cases of SCM type 2; only 7 patients had complication of syringomyelia. Thorough neurological examination, including muscle strength, sensation, pathological and physiological reflexes, was carried out in all of the patients. No apparent neurologic dysfunction was found before surgery. Only 1 patient complained of irregular urination, and thus underwent additional neural electromyography and urodynamic test. Based on all normal results of tests, this patient was also included in this study. The Ethics Committee of Xiangya Hospital of Central South University approved the study. All methods were performed in accordance with the relevant guidelines and regulations. Informed consent for study participation was obtained.

The indications for posterior-only surgical correction with heavy halo-femoral traction were based on the following criteria; (1) the patients suffered from CS associated with SCM; (2) coronal Cobb angle more than 60°; (3) the flexibility less than 30%; (4) consideration of the progression of the scoliosis.

Excluded criteria were based on; (1) SCM-related neurological symptoms were found before surgery, or neurological symptoms occurred during heavy halo-femoral traction; (2) complications with other complex intraspinal anomalies, including tethered cord, tumours, etc.; (3) short and sharp angular kyphoscoliosis, which required a osteotomy; (4) the patients that underwent any types of prophylactic neurosurgical intervention of SCM before.

### Preoperative traction

All patients underwent continuous preoperative heavy halo-femoral traction. The initial traction force applied from halo was 2 kg to the head and 2 kg through distal femur traction to their lower extremity. Every day, 2 kg traction force increased to the head and extremity respectively, if patients well tolerated. The maximum traction force could be 33 to 50% of the whole-body weight depending on patient’s tolerance. Neurological function was observed carefully during traction. The traction was applied for 18–20 h per day. The length of the traction period was mainly determined by the radiographic evidence of curve improvement on weekly radiographs. Traction continued till there was no significant improvement in Cobb angle on weekly radiographs [6, 7].

### Operative procedure

**Fig. 1:**
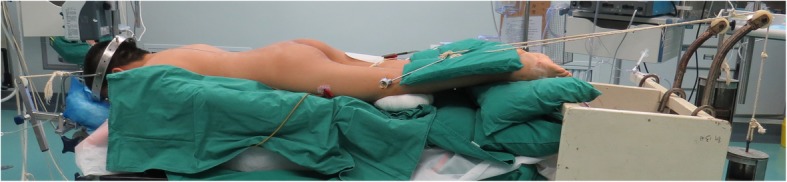
During the operation, heavy halo-femoral traction was maintained

During the operation, heavy halo-femoral traction was maintained (Fig. 1), and somatosensory evoked potential (SEP) and motor evoked potential (MEP) were thoroughly utilized to monitor the spinal cord functions. After exposure of posterior spinal components through a midline incision, pedicle screws (or hooks) were placed in the key vertebrae’s and adjacent to them for providing multiple anchor points. Facet joint capsules, intertransverse ligaments and contracture soft tissues at the rigid segments were released completely. Then, distraction, compression, rod rotation, and derotation should be adopted for correction. All structural curves need to be fixed and fused. Allogenous or autogenous bone grafts could be implanted for fusion [8–10].

### Postoperative procedure

After operation, the neurological examination was performed. Postoperatively, the patients were mobilized early and began to exercise 12 days later while wearing braces. All patients wore braces for an average of six months, and then gradually detached the braces.

### Scoliosis Research Society (SRS)-22 and modified Japanese Orthopaedic association (mJOA) scores

SRS-22 and mJOA scores were assessed preoperatively and at the final follow up to analyse clinical outcomes and neurological function. The SRS-22 questionnaire mainly included mental health, self-image, pain, and functional activities. Furthermore, bladder function, daily activities, clinical symptoms, and subjective symptoms were assessed by the mJOA score.

### Evaluation of radiography and statistical analysis

At the preoperative, postoperative, and final follow-up stages, the parameters of radiographs including coronal Cobb angle, flexibility ([preoperative Cobb angle – Cobb angle on the bending radiograph of the convex side]/preoperative Cobb angle), coronal Cobb angle after preoperative traction, correction rate were measured. The data was shown as means ± SD and analysed using SPSS 22.0. Paired t-test was used to compare the parameters preoperatively, postoperatively and at the final follow up. *P* < 0.05 indicates statistically significant difference.

## Results

The mean duration of surgery was 327.08 ± 43.99 min (range, 240–380 min) and the mean blood loss was 1303.33 ± 526.86 ml (range, 640–2100 ml). Thorough neurological examination was carried out in all of the patients after operation and at final follow up. No serious complications such as large vessel injury, spinal cord injury, or nerve injury occurred during operation. Moreover, there were no cases of cerebrospinal fluid leakage, death or deep infection, and none of the cases showed new irreversible neural injury.

The average period of follow up was 20.75 ± 8.29 months (range, 12–36 months). No complications related to instrumentation failure occurred. In one patient, the incisures of instrumentation were at the position higher than normal, which resulted in local pain and skin compression because of thinness.

### Correction

**Fig. 2:**
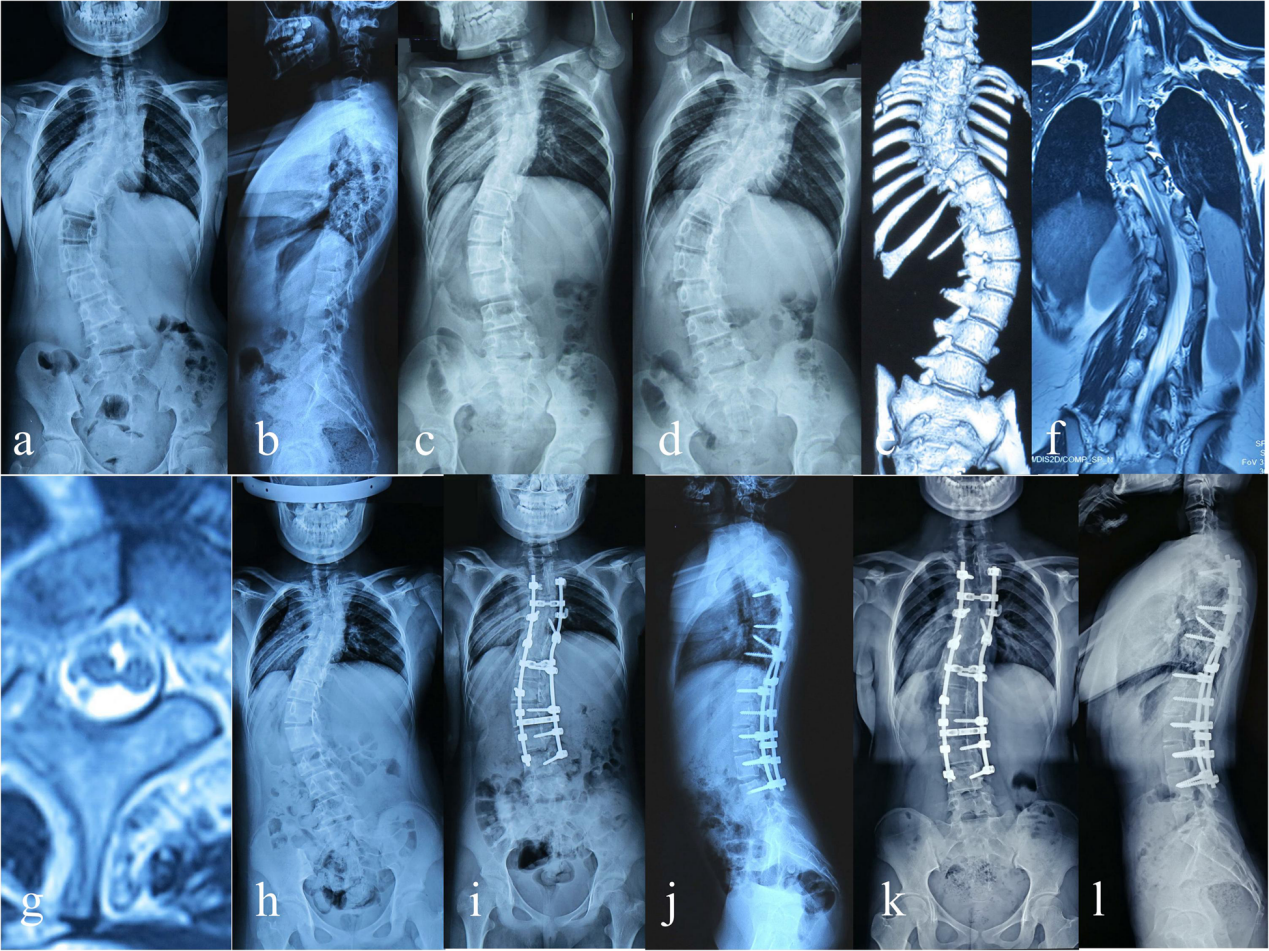
A patient with RCS accompanied by SCM. **a-b** Preoperative radiographs show that coronal Cobb angle was 62°.**c-d** Preoperative bending radiograph of the convex side shows that Cobb angle was 45.6° and flexibility was 26.45%.**e** Preoperative CT indicates mixed defects including failure of segmentation and formation. **f-g** Preoperative MRI indicates SCM type 2. **h** After heavy halo-femoral traction, the coronal Cobb angle was reduced to 38.2°.**i-j** After posterior-only surgical correction, postoperative radiographs show that coronal Cobb angle was 21.4°, and correction rate was 65.48%. **k-l** postoperative radiographs at 36 months after surgery show that coronal Cobb angle was 22.4°, and no signs of neurological impairment were found at the final follow-up stage

The preoperative mean coronal Cobb angle was 80.38° ± 13.55° (range, 60°-113°); on the bending radiograph of the convex side, the mean Cobb angle was 68.91° ± 15.48° (range, 44.5°- 98°); the mean flexibility was 15.04 ± 7.11% (range, 5.85–28.66%). After heavy halo-femoral traction, the mean coronal Cobb angle was reduced to 56.89° ± 13.39° (range, 35.6° - 87.5°), which showed statistically significant difference between the preoperative and post-traction data (*P* < 0.05). After posterior-only surgical correction, the postoperative mean coronal Cobb angle was further reduced to 32.54° ± 11.33° (range, 19.1° - 56.2°), which showed statistically significant difference between the post-traction and postoperative data (*P* < 0.05). The postoperative mean correction rate was 60.51 ± 7.79% (range, 48.19–69.40%). The coronal Cobb angle was 35.03° ±11.22° (range, 19.5° - 57.1°) at the final follow up, which revealed statistically significant difference between preoperative and the final follow-up data (*P* < 0.05), while the corrective loss rate of Cobb angle was only 3.17%. The patient’s trunk balance and body figure showed good improvement (Fig. 2) (Table 1).

**Table 1:** Preoperative, postoperative, and final follow-up measurement data

Case	Age (years)	Location of the major curve	AV	Period of follow up (months)	CS classification	SCM classification	Duration of surgery (min)	Blood loss (ml)	Pre-OP coronal Cobb (°)	Bending Cobb (°)	Flexibility (%)	After traction Cobb (°)	†Post-OP Cobb (°)	Post-OP correction rate (%)	‡Final follow-up Cobb (°)	Final follow-up correction rate (%)
1	11	L	L1/2	36	Mixed	Type 2	240	640	62	45.6	26.45	38.2	21.4	65.48	22.4	63.87
2	14	TL	L1	18	Mixed	Type 1	290	950	77	70	9.09	58	35	54.55	38.5	50
3	22	L	L2/L3	12	failure of formation	Type 2	365	1050	82.8	73.2	11.59	61.4	28.3	65.82	30.2	63.53
4	18	T	T10	12	failure of segmentation	Type 2	350	1600	81.2	70.3	13.42	64.2	25.5	68.6	32.9	59.48
5	24	T	T8	36	failure of segmentation	Type 1	380	2050	113	98	13.27	87.5	56.2	50.27	57.1	49.47
6	22	T	T7	36	failure of segmentation	Type 1	360	1960	94	88.5	5.85	62	48.7	48.19	49.9	46.91
7	10	L	L3	24	failure of formation	Type 2	269	750	60	44.5	25.83	35.6	19.1	68.17	19.5	67.5
8	17	T	T9	12	failure of segmentation	Type 1	365	1350	86	80	6.98	66.7	41	52.33	44.6	48.14
9	16	T	T6	18	failure of segmentation	Type 1	375	2080	91	82	9.89	63	45	50.55	47.8	47.47
10	15	TL	T12	24	Mixed	Type 2	290	820	67	47.8	28.66	40	20.5	69.4	23.6	64.78
11	11	L	L2	18	failure of segmentation	Type 1	350	1405	84.4	75.6	10.43	65.1	34.2	59.48	36.7	56.52
12	23	T	T7	18	failure of segmentation	Type 1	370	2100	105	91.4	12.95	76.3	49.8	52.57	53.2	49.33
13	18	T	T6	12	Mixed	Type 1	360	1865	89	82.5	7.3	64	42.8	51.91	44.2	50.34
14	10	L	L2	24	failure of segmentation	Type 2	250	790	69	51.6	25.22	38.3	22.4	67.54	24.8	64.06
15	13	T	T11	18	failure of formation	Type 1	310	998	74	66	10.81	55.2	29	60.81	31.7	57.16
16	17	T	T9	12	failure of segmentation	Type 1	340	1130	85	76	10.59	64	42	50.59	43.8	48.47
17	16	T	T8	18	failure of segmentation	Type 1	370	2020	96	85	11.46	69.4	47.3	50.73	49.4	48.54
18	15	L	L1/2	24	Mixed	Type 2	315	710	71	58.8	17.18	46.8	22.5	68.31	25.4	64.23
19	11	TL	T12/L1	24	failure of segmentation	Type 1	260	690	65	50.8	21.85	41.9	22.2	65.85	24.3	62.62
20	18	L	L2	12	failure of segmentation	Type 1	331	1780	87.3	74.2	15.01	65.3	34.2	60.82	36.8	57.85
21	24	L	L3	36	Mixed	Type 2	270	815	64	45.8	28.44	36.7	20.3	68.28	23.9	62.66
22	22	L	L2	12	failure of formation	Type 1	350	1797	78.4	66.5	15.18	55.9	24.1	69.26	26.5	66.2
23	12	TL	T12	24	Mixed	Type 2	360	1020	75.6	65.2	13.76	56.1	24.4	67.72	26.2	65.34
24	14	T	T10/T11	18	failure of segmentation	Type 2	330	910	71.4	64.5	9.66	53.8	25	64.99	27.4	61.62

Note: *AV* apical vertebrae, *CS* congenital scoliosis, *SCM* split cord malformation, *T* thoracic, *TL* thoracolumbar, *L* lumbar. The postoperative and preoperative data as well as the final follow-up and preoperative data were analyzed using paired t tests. *P* < 0.05 implies statistically significant difference. † *P* < 0.05 (postoperative vs. preoperative); ‡ *P* < 0.05 (final follow-up vs. preoperative)

### SRS-22 score

SRS-22 scores were assessed preoperatively and at the final follow up. The mental health, self-image, pain, and functional activities scores revealed good improvement at the final follow up (Table 2), especially the mental health and self-image scores (*p* < 0.05). At the final follow up, the total score increased from 67.22 ± 5.54 to 84.57 ± 4.71. It revealed significant statistical differences between final follow-up and preoperative scores (*p* < 0.01).

**Table 2:** SRS-22 score of preoperative and final follow up

Parameters	Preoperative	Final follow-up	T value	*P* value
Functional activity	17.60 ± 2.46	20.68 ± 1.53	−5.208	0.000
Pain	20.75 ± 1.94	21.98 ± 1.86	−2.240	0.007
Self image	14.77 ± 2.48	19.16 ± 2.28	−6.384	0.000
Mental health	15.06 ± 2.15	19.16 ± 1.92	−6.368	0.000
OP Satisfaction	–	7.94 ± 1.34	–	–
SRS-22 total score	67.22 ± 5.54	84.57 ± 4.71	−11.689	0.000

(Note: SRS-22 questionnaires including five aspects: 1. Recovery of functional activities of patients include question 5, 9, 12, 15, 18; 2. Improvement of pain of the patients include question 1, 2, 8, 11, 17; 3. Assessment of self image of the patients include question 4, 6, 2, 14, 19; 4. Assessment of mental health of the patients include question 3, 7, 13, 16, 20; 5. Operation satisfaction was only answered by patients performed operation include question 21, 22)

### mJOA score

Neurological function was evaluated by mJOA score. There were no significant differences between final follow-up and preoperative scores (p>0.05). At the final follow up, the total score increased from 26 ± 2.2 to 27 ± 1.9. Bladder function, daily activities, clinical symptoms, and subjective symptoms showed no significant differences between final follow-up and preoperative evaluation (*p* = 0.669, *p* = 0.496, *p* = 0.942, *p* = 0.067; Table 3).

**Table 3:** mJOA score of preoperative and final follow up

Parameters	Preoperative	Final follow-up	T value	*P* value
Subjective symptom	8.1 ± 1.1	9.0 ± 0.6	−1.980	0.067
Clinical symptom	6.3 ± 0.2	6.3 ± 0.4	0.242	0.942
Daily activities	12.9 ± 1.0	13.1 ± 0.9	−0.686	0.496
Bladder function	−0.4 ± 0.9	− 0.5 ± 0.7	0.430	0.669
mJOA total score	26 ± 2.2	27 ± 1.9	−1.685	0.099

Note: Total mJOA-score was 29 including subjective symptom from 0 to 9 score, clinical symptom from 0 to 6 score, daily activities from 0 to 14 score and bladder function from - 6 to 0 score

## Discussion

Somites, which vertebrae derived from, enclosed neural tube during embryonic development. Any injury resulting in vertebral deformity during embryonic development may cause neural tube defects. Therefore, CS is usually accompanied by intraspinal anomalies, including SCM, tethered cord, syringomyelia, Arnold Chiari malformation, etc. There may also be multiple intraspinal anomalies at the same time [11, 12]. In fact, CS and SCM usually occurred simultaneously in clinical practice. According to report, SCM was observed in 4.0 to 9.0% of patients with CS. The dorsolumbar and lumbar regions are the most common sites. The clinical symptoms could be summarized as following characteristics: lower extremity weakness, atrophy, and deformity, scoliosis, spinal bifida, skin lesions, sphincter dysfunction [13, 14].

However, most of outpatients with CS accompanied by SCM showed no signs of neurological impairment. Patients often presented with spinal deformity during their first visit to the doctor, and SCM was discovered only by chance in the examination of CT and MRI. Furthermore, the spinal cord and Dural sac may be compressed by a bony or fibrocartilaginous spur of SCM during orthopaedic surgery for CS associated with SCM, which causes neurologic injury postoperatively. The presence of SCM greatly increases the risks of correction in patients with CS.

Regarding CS accompanied by SCM, the main purpose of surgery is not only to correct spinal deformity and prevent progression of deformity, but also to prevent nerve injury. Thus far, the neurosurgical management of a bony or fibrocartilaginous spur of SCM before undertaking corrective surgery and corrective procedures are still controversial. Ayvaz et al. [15] advised that neurosurgical interventions (spur excision and dural reconstruction) should be recommended even for neurologically asymptomatic SCM type 1 before the corrective surgery to the CS, whereas patients with SCM type 2 can be treated safely without a need of neurosurgical intervention. In SCM Type 2, it allows the spinal cord to move relatively independently in the spinal canal, because two hemi cords exist in one dural canal without substantial spur. Therefore, there is no need for additional canal work.

Some authors [16, 17] advocated that the approach for management of CS associated with SCM was first to perform surgery for SCM and then to perform orthopaedic surgery for correction of the spinal deformity, approximately 3 to 6 months later. The aim was to prevent spinal cord injury during deformity correction and reduce the incidence of postoperative complications of the neural system. However, there are several disadvantages of staged procedures: (1) Due to less clear anatomic landmarks, surgically complex exposure, and more blood loss, the follow-up correction becomes more difficult and complicated. In addition, at the surgical site, a preformed adhesion and possible retethering could make complex reconstructive operations such as osteotomy more difficult. (2) The patients suffer from the risks of anaesthesia and surgery more than once. (3) The staged procedures increase the financial and psychological burden in patients, and prolong hospitalization and rehabilitation.

In recent years, Hui et al. [18] have reported one-stage operation was effective and safe for the treatment of CS and SCM, but resection of bony spur was still recommended. In these cases, the correction rate of main curve was 54.5% without increasing complications. However, neurosurgical intervention itself is characterized with high risk of operation and neurological complications. For surgical interventions of SCM alone, the risk of infection, cerebrospinal fluid leakage, and neurological deterioration after neurological intervention was approximately 7 to 31% [14, 19]. Therefore, Feng et al. [5] compared the results of two surgical strategies for the treatment of SCM type 1 and CS. In the resection of bony spur (BR) group, the neurological complications, blood loss, and duration of surgery were significantly higher than those in the nonresection of bony spur (NR) group. Moreover, prophylactic neurosurgical intervention before corrective surgery was probably not necessary in patients with stable or intact neurological function.

Posterior spine-shortening osteotomy has recently been developed [3, 4]. The correction of the scoliosis is performed following the osteotomy. The scoliosis is corrected later by shortening and compression in the vertebrectomy gap, which relieves the longitudinal tension. Theoretically, it should prevent the spinal cord from stretch injury. However, the correction of the scoliosis in the presence of the spinal cord being tensioned by the bony or fibrocartilaginous spur in SCM still poses significant risks. Therefore, one-stage operation including resection of bony spur and subsequent spine-shortening osteotomy was recommended for preventing spur-related complications. Nevertheless, this method presented new challenges, such as more frequent neurological complications, high level of technical requirements, difficult operation, blood loss, and operation time at a single operation. Furthermore, neurosurgical interventions (spur excision and Dural reconstruction) were still performed at the same time, and there were neurosurgical complications related to spur.

24 patients, suffered from RCS associated with SCM, were treated at our Department. No apparent neurologic dysfunction was found in all patients. All patients underwent continuous preoperative heavy halo-femoral traction, with gradual initial traction monitoring the neurological function carefully; lengthening the spine step by step. During the surgery, facet joint capsules and contracture soft tissues were released completely and widely without spur excision. After posterior-only surgical correction, the postoperative mean correction rate was 60.51%. The patients’ body figure and trunk balance showed good improvement. This correction rate was higher than that of spine-shortening osteotomy reported by some authors [3, 18], and was similar to that of Chen’s osteotomy [4]. However, the operation time, blood loss, difficulty of operation and incidence of neurological complications were significantly lower than those reported in the literature [4, 18].

Therefore, our findings can be summarized in the following characteristics: (1) With initial skeletal traction gradually, the preoperative traction may increase the tolerance of spinal cord to stretch trees and ischemia from correction of curve. The patient’s neurological status was frequently checked and assessed on preoperative bending and suspension position, and heavy halo-femoral traction, so as to provide a basis for intraoperative orthopaedic procedures, and diminish risks of neurological complications. (2) The preoperative heavy halo-femoral traction may significantly improve curve flexibility and spinal compliance, which allowed for a better overall correction. (3) For neurologically asymptomatic SCM, prophylactic neurosurgical intervention (spur excision and dural reconstruction) itself was characterized by increasing risk of operation and neurological complications. (4) Posterior-only surgical correction with heavy traction was performed to avoid the risks of anaesthesia and surgery more than once, and the disadvantages of spine-shortening osteotomy, such as more operation time, blood loss, difficult operation, high level of technical requirements, and frequent neurological complications. (5) The scoliosis was rigid in all the patients of this study, and the flexibility was only 15.04%. During the surgery, facet joint capsules and contracture soft tissues should be released completely and widely to increase spinal flexibility. (6) Neurological monitoring (SEP and MEP) was the guarantee of the whole operation. (7) If neurological symptoms were found before surgery, or neurological symptoms occurred during heavy traction, our method was not appropriate for these patients, who needed the prophylactic neurosurgical intervention of SCM.

## Conclusions

In conclusion, without prophylactic neurosurgical intervention and spine-shortening osteotomy, posterior-only surgical correction with heavy halo-femoral traction could be safe and effective for the treatment of RCS associated with SCM in decreasing the incidence of complications. However, before any procedure, appropriate surgical interventions must be chosen carefully; adequate monitoring during the surgery, preoperative heavy traction and evaluation are necessary to improve clinical results.

## Data Availability

The datasets analyzed during the current study are not publicly available because a further study about SCM is in progress in our institution, but are available from the corresponding author on reasonable request.

## Abbreviations

CS: Congenital scoliosis
CT: Computed tomography
MEP: Motor evoked potential
mJOA: Modified Japanese Orthopaedic Association
MRI: Magnetic resonance imaging
RCS: Rigid congenital scoliosis
SCM: Split cord malformation
SEP: Somatosensory evoked potential
SRS: Scoliosis Research Society
